# Molecular Epidemiology of Influenza A/H3N2 Viruses Circulating in Uganda

**DOI:** 10.1371/journal.pone.0027803

**Published:** 2011-11-21

**Authors:** Denis K. Byarugaba, Mariette F. Ducatez, Bernard Erima, Edison A. Mworozi, Monica Millard, Hannah Kibuuka, Luswa Lukwago, Josephine Bwogi, Blanche B. Kaira, Derrick Mimbe, David C. Schnabel, Scott Krauss, Daniel Darnell, Richard J. Webby, Robert G. Webster, Fred Wabwire-Mangen

**Affiliations:** 1 Faculty of Veterinary Medicine, Makerere University, Kampala, Uganda; 2 Makerere University Walter Reed Project, Kampala, Uganda; 3 Department of Infectious Diseases, St. Jude Children's Research Hospital, Memphis, Tennessee, United States of America; 4 College of Health Sciences, Makerere University, Kampala, Uganda; 5 Ministry of Health, Kampala, Uganda; 6 Uganda Virus Research Institute, Entebbe, Uganda; 7 United States Army Research Unit, Nairobi, Kenya; Centers for Disease Control and Prevention, United States of America

## Abstract

The increasing availability of complete influenza virus genomes is deepening our understanding of influenza evolutionary dynamics and facilitating the selection of vaccine strains. However, only one complete African influenza virus sequence is available in the public domain. Here we present a complete genome analysis of 59 influenza A/H3N2 viruses isolated from humans in Uganda during the 2008 and 2009 season. Isolates were recovered from hospital-based sentinel surveillance for influenza-like illnesses and their whole genome sequenced. The viruses circulating during these two seasons clearly differed from each other phylogenetically. They showed a slow evolution away from the 2009/10 recommended vaccine strain (A/Brisbane/10/07), instead clustering with the 2010/11 recommended vaccine strain (A/Perth/16/09) in the A/Victoria/208/09 clade, as observed in other global regions. All of the isolates carried the adamantane resistance marker S31N in the M2 gene and carried several markers of enhanced transmission; as expected, none carried any marker of neuraminidase inhibitor resistance. The hemagglutinin gene of the 2009 isolates differed from that of the 2008 isolates in antigenic sites A, B, D, and to a lesser extent, C and E indicating evidence of an early phylogenetic shift from the 2008 to 2009 viruses. The internal genes of the 2009 isolates were similar to those of one 2008 isolate, A/Uganda/MUWRP-050/2008. Another 2008 isolate had a truncated PB1-F2 protein. Whole genome sequencing can enhance surveillance of future seasonal changes in the viral genome which is crucial to ensure that selected vaccine strains are protective against the strains circulating in Eastern Africa. This data provides an important baseline for this surveillance. Overall the influenza virus activity in Uganda appears to mirror that observed in other regions of the southern hemisphere.

## Introduction

Clinical surveillance and genetic analysis are invaluable in guiding effective influenza control measures. In sub-Saharan Africa, however, most outbreaks of influenza go unreported and information about circulating strains is relatively limited [1]. Partial sequences and strain information are currently available from only a few countries in Africa which increased their surveillance efforts after the emergence of influenza A, H5N1, in humans in 2003 [2], [3]. The 2009 H1N1 influenza pandemic underscored the importance of surveillance networks that can rapidly characterize circulating viruses for effective response and containment [4].

Influenza A viruses evolve rapidly; this characteristic allows them to regularly generate new strains to which human immunity is lacking, thereby causing periodic pandemics [5]. Of the 16 known subtypes of hemagglutinin (HA) and nine subtypes of neuraminidase (NA) in influenza A viruses [6] only subtypes H3N2 and H1N1 currently circulate in the human population. In Sub-Saharan Africa, H3N2 was the predominant subtype in 2009, when the swine-origin influenza A (H1N1) virus was introduced into the human population [7]. The two virus subtypes are currently co-circulating [8].

Minor mutations in the eight gene segments of influenza A viruses, especially the HA segment, can alter viral antigenic epitopes sufficiently to evade immune recognition (antigenic drift) [9]. In some cases, different viruses co-circulating in the same host can exchange entire gene segments, resulting in re-assortment of the genome and generating new virus strains (antigenic shift) [10]. These changes can produce more fit viruses that cause new epidemics or pandemics. Vaccination is the principal strategy to reduce the public health burden of influenza, but its effectiveness depends on influenza surveillance and full genome analysis of the viruses isolated [11]. Although influenza vaccination is very limited in most of Africa, particularly Sub-Saharan Africa, viruses that have undergone vaccination pressure are frequently introduced from other regions [12].

Most influenza sequencing has focused on the HA1 domain of the hemagglutinin gene, where mutations have the greatest effect on antigenic structure [7]. However, sequencing of the whole influenza virus genome facilitates comparison and understanding of the evolutionary dynamics of circulating viruses and the prediction of potential evolution events that are likely to result in new strains [13]. It also allows closer examination of the importance of other genes in influenza outbreaks and vaccine selection. A detailed examination of the whole genomes of some recent H3N2 viruses revealed that multiple lineages can co-circulate, persist, and re-assort in epidemiologically significant ways that are not easily discerned by examining the HA genes alone [14], [15]. Holmes and colleagues [15] demonstrated that some H3N2 isolates could not be distinguished on the basis of their HA genes but could be assigned to different clades on the basis of their seven other gene segments. Therefore, whole genome sequencing allows better monitoring of evolutionary events that can predict the emergence of viruses with pandemic potential (Fricke [16], [17] and subsequently provides vital data on global viral spread better informing control strategies. Here, we describe the whole genome analysis of influenza A/H3N2 viruses isolated in Uganda during the 2008 and 2009 seasons and compare these viruses with other African isolates and with the pertinent WHO reference vaccine strains.

## Materials and Methods

### Sample collection

Samples were collected at two Ugandan sentinel surveillance sites established in 2008: Mulago National Referral Hospital in Kampala and Kayunga District Hospital in Kayunga District. Nasopharyngeal and throat samples were collected from individuals 6 months or more of age with influenza-like symptoms at the two facilities' outpatient clinics from October 1, 2008 through September 30, 2009. Inclusion criteria were fever (≥38°C) plus either cough or sore throat within the past 72 hours prior to patient presentation. Any patient too ill to participate or unwilling or unable to provide consent to participate in the surveillance study was excluded. All participants/caretakers were informed about the study and consented to participate in the study by signing (or by a thumbprint if unable to write) a consent form if 18 yrs or older signed. For children aged less than 18 yrs, their parents/guardians signed the informed consent form while for minors aged 8 to 17 yrs, they together with their parents/guardians signed the consent form. Nasopharangeal and throat swab samples were collected using a dacron swab in a 2-ml cryovial containing virus transport medium. After collection, the samples were immediately stored at −196°C in a liquid nitrogen dry shipper or kept on ice if the samples were to be delivered to the laboratory in less than 8 hours. All samples were transported to Makerere University Walter Reed Influenza Research Laboratories (daily from Mulago Hospital and within one week from Kayunga Hospital). Sample collection at these surveillance sites began in October 2008, and specimens analyzed for this study were collected from this time through September 2009. The study was approved by the Makerere University School of Public Health Institutional Review Board, the US Army Research and Material Command, and the Uganda National Council for Science and Technology.

### Influenza A screening by RT-PCR

Viral RNA was extracted from all samples by using the QIAamp Viral RNA mini kit (Qiagen) according to the manufacturer's directions. RT-PCR of the extracts was performed by using a Qiagen One-Step RT-PCR kit according to the manufacturer's instructions, with the following influenza A matrix gene primers M52C forward: 5′CTTCTAACCGAGGTCGAAACG-3′ and M253R reverse: 5′-AGGGCATTT TGGACAAAKCGTCTA-3′ (TAGc, Copenhagen) as described [18]. (Screening for flu B was simultaneously done by Flu-B M-gene specific primers but data is not included in this paper except the relative occurrence). Briefly, the 25-µl reaction volume contained 5 µl of 5× PCR buffer, 13 µl of RNAse-free H_2_0, 1 µl of 10 mmol/L dNTPs, 1.5 µl of 10 nmol/L reverse primer, 1.5 µl of 10 nmol/L forward primer, 1 µl of enzyme mix (Taq DNA polymerase and reverse transcriptase), and 2 µl of viral RNA extract. Amplification was carried out in an Applied Biosystems Veriti 96-well thermocycler with a single reverse transcription step of 50°C for 30 min, “hot start PCR” (95°C) for 15 sec, forty 30-sec denaturation cycles at 95°C, 30 sec of primer annealing at 55°C, 1 min of extension at 72°C, and further extension for 10 min at 72°C. The samples (including a known positive control) were then separated on a 1% agarose gel with a 50-bp marker. The primers amplified a 250-bp segment of the matrix gene in influenza A–positive samples; this product was visualized and documented in a Biorad Gel Doc XR imager.

### Virus isolation and subtyping

PCR-positive samples (100 µl) were inoculated on 70%–90% confluent Madin-Darby Canine Kidney cell line (MDCK) (NBL2; American Type Culture Collection,(ATCC) Rockville, Md.) in flat-sided tubes after pre-treatment with TPCK trypsin to facilitate virus entry. Tubes were capped loosely, incubated in a tissue culture incubator at 37°C with 5% CO_2_, and observed daily for 10 days for cytopathic effects by light microscopy using an inverted microscope. When cytopathic effects were observed, 10 µl of supernatant fluid was placed in the chamber of a multiwell slide and allowed to dry. Influenza virus was confirmed by immunofluorescence assay with antibodies against influenza A or B. The isolates were subtyped by RT-PCR using primers specific for H3, H1, (from CDC) and N1, and N2 as previously described [19]. All isolates were stored at −80°C until further characterization.

### Sequencing and sequence analysis

The influenza H3N2 isolates isolated during this period were sequenced at the WHO Collaborating Center for the Ecology of Influenza in Animals and the NIAID Center of Excellence in Influenza Research and Surveillance (CEIRS) at St. Jude Children's Research Hospital, Memphis, TN. Isolates were sequenced by using next-generation DNA technologies (Illumina Genome Analyzer) according to the manufacturer's instructions. To amplify all eight segments in a single reaction, RT-PCR was conducted on RNA templates using Uni-12, Uni-13, and polymerase gene primers as previously described [20] plus Invitrogen SuperScript III One-Step Reverse Transcriptase and Platinum Taq HiFi (Invitrogen). Sanger sequencing was carried out to fill out some of the sequences gaps remaining after illumina sequencing at the Hartwell Center of St. Jude Children's Research Hospital, Memphis, TN.

**S**equences were aligned using the Bioedit program [21]. The eight gene segments were phylogenetically analyzed on the basis of their nucleotide/protein sequences. The MEGA version 4.0 program [22] was used for tree building by the neighbor-joining method. The number of bootstrap replications was set to 1,000, and bootstrap values above 50 were labeled on major tree branches for reference. The Ugandan virus strains were clustered on the basis of nucleotides, and only dominant clusters were used to infer phylogenetic relationships. The analysis included sequences from all relevant African human virus isolates available in GenBank and GISAID databases and the sequences of H3N2 vaccine strains recommended by the World Health Organization for the 2007, 2008, and 2009 seasons (A/Brisbane/10/2007, A/Victoria/208/2009, A/Perth/16/2009).

#### Accession numbers

The genebank sequence accession numbers for the 444 segments of the 59 isolates included in this study are from CY087308 though to CY087751.

## Results

### Patients

**Figure 1:**
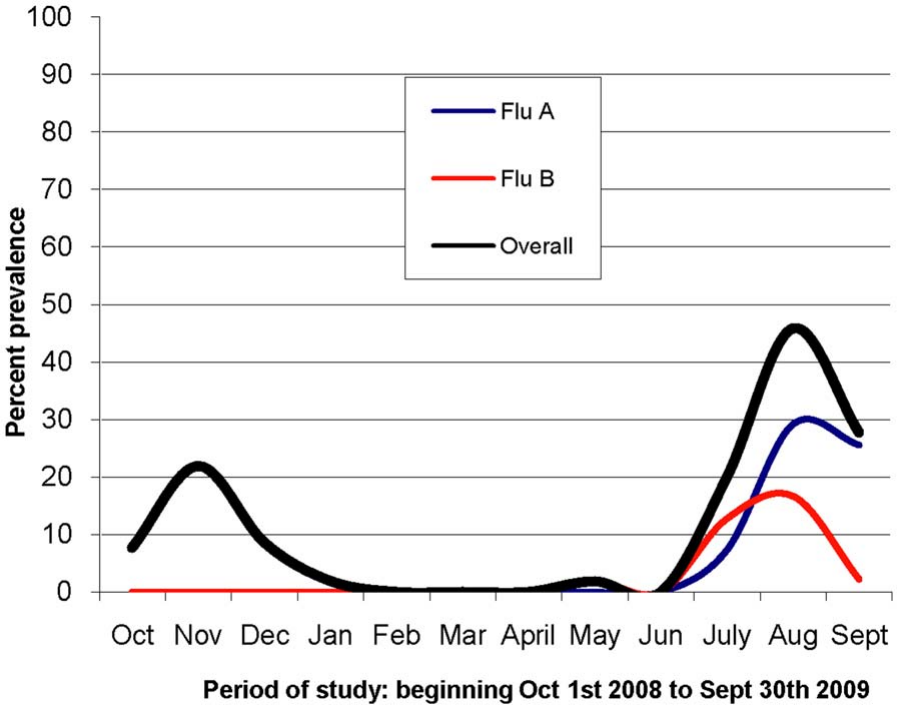
Distribution of influenza cases from October 1, 2008 through September 30, 2009. The figure shows the two influenza seasons. The 2008 season was only Flu A and peaked in November while the 2009 season was both FluA and B and peaked in August. Flu A was more prevalent than Flu B. This paper discusses only Flu A.

During the study period, 932 samples were collected from consenting patients at the two sentinel sites (733 at Mulago Hospital and 199 at Kayunga Hospital). The majority (77%) of study participants were ≤5 years of age (Table 1). The participants from whom the samples were collected were all outpatients presenting with a fever (≥38°C) plus either cough or sore throat within the past 72 hours prior to patient presentation. The rate of influenza A virus recovery was 7.9% (72 isolates). The distribution of cases followed the typical tropical seasonal pattern, peaking during July, August, and September (Fig. 1). The present study focused on the H3N2 subtype of influenza A. Of the 59 samples positive for H3N2 influenza virus, only five were from Kayunga Hospital (all collected in 2008).

**Table 1:** Distribution of study participants according to age and sample site.

Age group(years)	Mulago National Referral Hospital: n (%)	Kayunga District Hospital: n (%)	Total
0–5	657 (89.6%)	69 (34.7%)	726 (77%)
6–17	49 (6.7%)	25 (12.6%)	74 (8%)
18–20	5 (0.7%)	30 (15.1%)	35 (4%)
21–30	6 (0.8%)	27 (13.6%)	33 (4%)
31–40	1 (0.1%)	25 (12.6%)	26 (3%)
>40	0 (0%)	19 (9.5%)	19 (2%)
Not recorded	15 (2.0%)	4 (2.0%)	19 (2%)
**Total**	**733**	**199**	**932**

### Sequence analysis

A total of 444 gene segments from the 59 isolates were analyzed. Table 2 lists the number of specific gene segments of the 59 isolates (51 isolates from 2008 and 8 from 2009) that were sequenced. Nucleotide and amino acid sequences for the whole genome were compared within the Uganda isolates and between these isolates, the WHO vaccine strains and available sequences of other African isolates. The 2008 and 2009 isolates had differences in all eight gene segments or their encoded proteins. The HA, NA, nucleoprotein (NP), nonstructural protein 1 (NS1), matrix 1 (M1), and matrix 2 (M2) genes as well as the nonstructural protein 2 (NS2), polymerase acidic (PA), polymerase basic 1 (PB1), PB1-F2, and PB2 protein sequences also clearly differentiated the two 2008 and 2009 seasons. However, all of the 2009 isolates were similar to one 2008 isolate: A/Uganda/MUWRP-050/2008 (H3N2) for all but HA genes. Kimura genetic distances between the 2008 isolates and the 2009 isolates ranged from 0.7% to 1.7% at the nucleotide level and from 1.7% to 6% at the amino acid level (Table 2).

**Table 2:** Genetic pair-wise distance matrix for the Ugandan isolates.

Gene/Protein (no. of sequences analyzed)	Maximum Kimura 2P genetic distance (nt level), %	Maximum Poisson correction genetic distance (aa level), %
PB2 (53)^1^	1.1	1.6
PB1 (57)^1^	1.7	1.8
PB1-F2 (49)	-	6.0
PA (58)^1^	1.0	1.4
HA (59)	1.2	1.7
NP (59)	0.7	0.4
NA (59)	0.9	1.3
NS (57)	1.2	-
NS1 (57)	-	2.0
NS2 (57)	-	2.0
M (59)	0.7	-
M1 (59)	-	0.8
M2 (59)	-	2.1

1 Partial sequences were included in the analysis of PB2, PB1, and PA genes.

### Surface glycoprotein genes and gene products

#### HA gene

**Figure 2:**
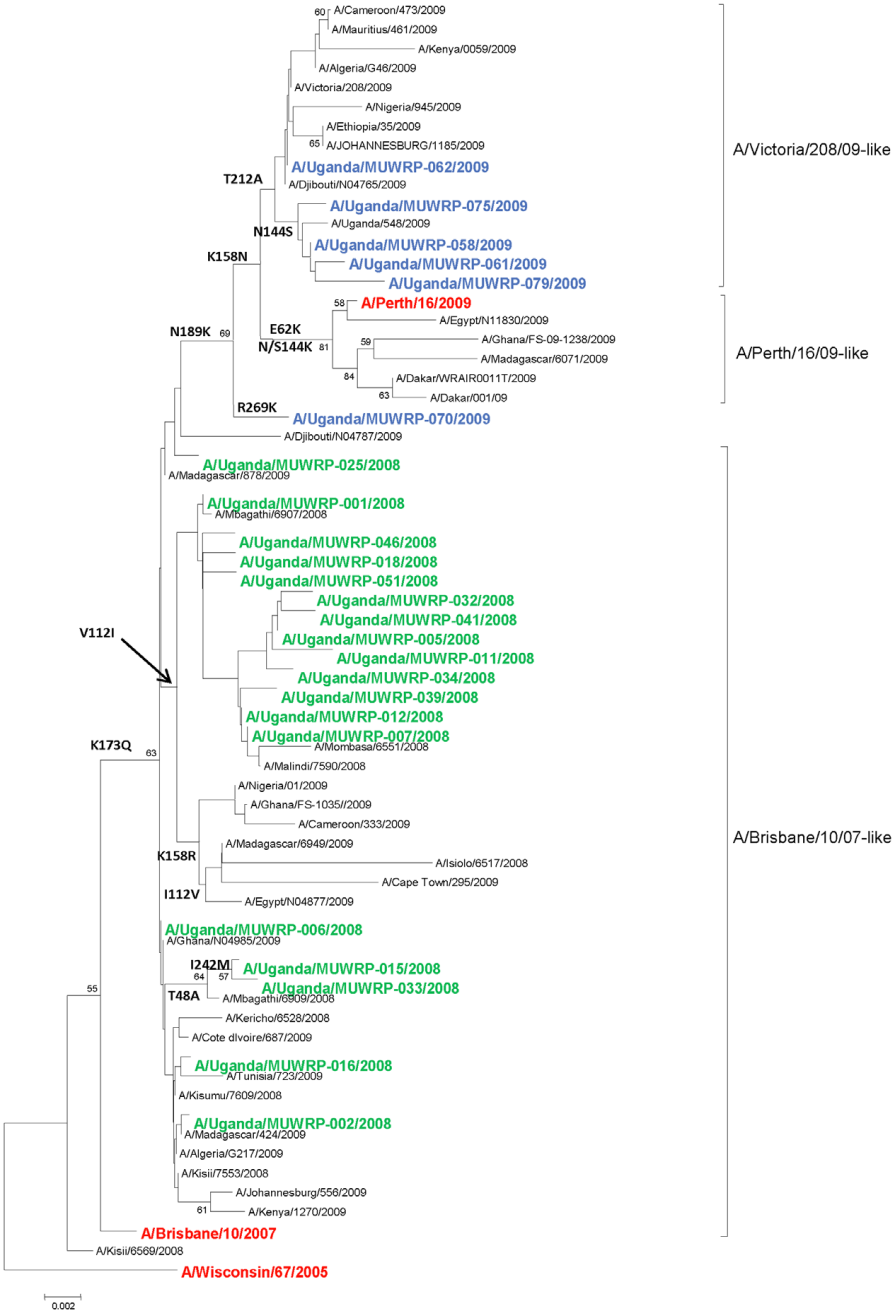
Phylogenetic analysis of the influenza A/H3N2 HA genes. The phylogenetic tree shows clustering of the Ugandan isolates into two distinct major clades corresponding to the year of isolation. The 2008 isolates clustered with the A/Brisbane/10/2007/-like virus clade, while the 2009 isolates clustered with the A/Victoria/208/09-like virus clade, with the exception of one isolate. Also included in the tree are the last 3 recommended H3N2 vaccine strains (A/Wisconsin/67/2005, A/Brisbane/10/2007, and A/Perth/16/2009, in red font) and other relevant African isolates (at least 1 sequence per country, but excluding identical sequences at the aa level). Ugandan isolates from 2008 and 2009 are shown in green and blue, respectively. A single strain was kept as a representative of identical sequences, hence there are less than 59 strains represented on the tree. At the amino acid level, the HA sequences of A/Uganda/MUWRP-03/2008, A/Uganda/MUWRP-08/2008, A/Uganda/MUWRP-09/2008, A/Uganda/MUWRP-10/2008, A/Uganda/MUWRP-13/2008, A/Uganda/MUWRP-14/2008, A/Uganda/MUWRP-17/2008, A/Uganda/MUWRP-19/2008, A/Uganda/MUWRP-21/2008, A/Uganda/MUWRP-23/2008, A/Uganda/MUWRP-26/2008, A/Uganda/MUWRP-27/2008, A/Uganda/MUWRP-28/2008, A/Uganda/MUWRP-45/2008, and A/Uganda/MUWRP-49/2008 were indeed identical to A/Uganda/MUWRP-01/2008 present on the tree; A/Uganda/MUWRP-20/2008 and A/Uganda/MUWRP-38/2008 to A/Uganda/MUWRP-05/2008; A/Uganda/MUWRP-22/2008, A/Uganda/MUWRP-24/2008, A/Uganda/MUWRP-29/2008, A/Uganda/MUWRP-42/2008, A/Uganda/MUWRP-43/2008, A/Uganda/MUWRP-44/2008, A/Uganda/MUWRP-48/2008, A/Uganda/MUWRP-50/2008, and A/Uganda/MUWRP-52/2008 to A/Uganda/MUWRP-06/2008; A/Uganda/MUWRP-40/2008 to A/Uganda/MUWRP-07/2008; A/Uganda/MUWRP-31/2008 to A/Uganda/MUWRP-15/2008; A/Uganda/MUWRP-35/2008, A/Uganda/MUWRP-36/2008, and A/Uganda/MUWRP-37/2008 to A/Uganda/MUWRP-34/2008; A/Uganda/MUWRP-47/2008 to A/Uganda/MUWRP-46/2008; and A/Uganda/MUWRP-65/2009 and A/Uganda/MUWRP-86/2009 to A/Uganda/MUWRP-58/2009.

The HA coding sequences and amino acid sequences of the 59 Ugandan isolates were analyzed. All of the HAs showed the K73Q amino acid (aa) substitution that was characteristic of the A/H3N2 viruses circulating in most parts of the world at that time as evidenced by the phyologenetic comparison to WHO vaccine strain and the other selected publicly available sequences (Figure 2). Phylogenetic analysis showed the isolates to cluster into two distinct major clades corresponding to the year of isolation. The 2008 isolates clustered with the A/Brisbane/10/2007 vaccine strain, while the 2009 isolates clustered with the A/Victoria/208/2009-like viruses with the exception of one isolate, A/Uganda/MUWRP-070/2009 (Figure 2). The 2009 isolates differed from the 2008 isolates at two antigenic sites: antigenic site B, which contained an N189K aa substitution, and antigenic site D, which showed a T212A substitution. The 2009 isolates also had other aa substitutions at antigenic site B: N144S (with the exception of A/Uganda/MUWRP-070/2009 and A/Uganda/MUWRP-062/2009) and K158N (with the exception of A/Uganda/MUWRP-070/2009). In addition, all of the 2009 isolates had a single silent cytosine–to-adenine nucleotide substitution at position 270, although this mutation did not result in an amino acid change. A/Uganda/MUWRP-070/2009 differed from all other isolates in this study in having an R269K aa substitution, and A/Uganda/MUWRP-079/2009 differed from all other isolates in having an I192T and an I245V aa substitution.

All of the isolates had the classical receptor binding site motif with 19Y, 136S, 153W, 183H, 195Y, and 225–228 NIPS in the HA protein, although as expected, there were differences in the antigenic sites. The major differences between the 2008 and 2009 isolates were at antigenic sites A, B, and D. At antigenic site A, Thirty eight of the 2008 isolates had the IRRSNNS motif, while the all the others had the IRRSSNS motif. At antigenic site B, the 2008 isolates had the THLKFK motif, while the 2009 isolates had the THLNFK motif; at antigenic site D, the 2008 isolates had the VSTKRSQQTVIPNIGSR motif, while the 2009 isolates had the VSTKRSQQAVIPNIGSR motif.

#### NA gene

**Figure 3:**
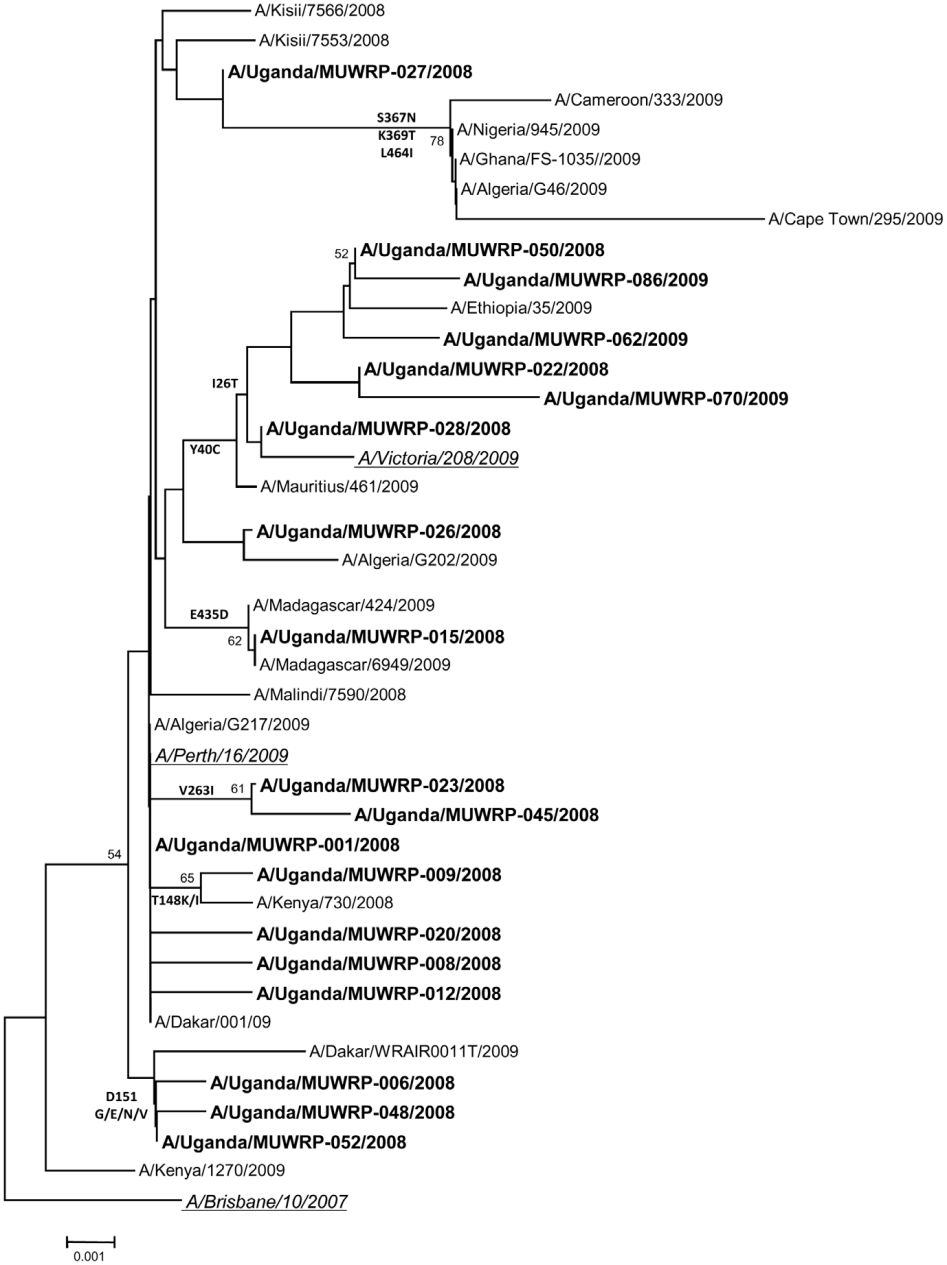
Phylogenetic analysis of the influenza A/H3N2 NA genes. The phylogenetic tree shows two lineages that differentiate the 2009 and 2008 isolates, although all had the D147N and I215V genetic characteristics of the A/Brisbane/10/2007-like viruses. The NA genes of the 2009 isolates (in blue) differed from those of the 2008 isolates (in green) by two amino acid substitutions: I26T and Y40C. Vaccine strains A/Brisbane/10/2007 and A/Perth/16/2009 are shown in red. Relevant African isolates (at least 1 sequence per country, but excluding identical sequences at the aa level) were also included. A single strain was kept as a representative of identical sequences, hence there are less than 59 strains represented on the tree. At the amino acid level, the NA sequences of A/Uganda/MUWRP-02/2008, A/Uganda/MUWRP-03/2008, A/Uganda/MUWRP-05/2008, A/Uganda/MUWRP-07/2008, A/Uganda/MUWRP-10/2008, A/Uganda/MUWRP-11/2008, A/Uganda/MUWRP-13/2008, A/Uganda/MUWRP-14/2008, A/Uganda/MUWRP-16/2008, A/Uganda/MUWRP-18/2008, A/Uganda/MUWRP-21/2008, A/Uganda/MUWRP-22/2008, A/Uganda/MUWRP-24/2008, A/Uganda/MUWRP-27/2008, A/Uganda/MUWRP-28/2008, A/Uganda/MUWRP-29/2008, A/Uganda/MUWRP-32/2008, A/Uganda/MUWRP-34/2008, A/Uganda/MUWRP-35/2008, A/Uganda/MUWRP-36/2008, A/Uganda/MUWRP-37/2008, A/Uganda/MUWRP-38/2008, A/Uganda/MUWRP-40/2008, A/Uganda/MUWRP-41/2008, A/Uganda/MUWRP-42/2008, A/Uganda/MUWRP-43/2008, A/Uganda/MUWRP-44/2008, A/Uganda/MUWRP-46/2008, A/Uganda/MUWRP-47/2008, A/Uganda/MUWRP-49/2008, A/Uganda/MUWRP-51/2008, and A/Uganda/MUWRP-52/2008 were indeed identical to A/Uganda/MUWRP-01/2008 present on the tree; A/Uganda/MUWRP-22/2008, A/Uganda/MUWRP-58/2009, A/Uganda/MUWRP-61/2009, A/Uganda/MUWRP-65/2009, A/Uganda/MUWRP-74/2009, A/Uganda/MUWRP-75/2009, and A/Uganda/MUWRP-79/2009 to A/Uganda/MUWRP-50/2008.

Phylogenetic analysis of the NA genes of the 59 Ugandan isolates also indicated two lineages that clearly differentiated the 2008 and 2009 isolates (Figure 3). All isolates were descended from A/Brisbane/10/2007-like viruses, with the D147N and I215V genetic characteristics. The NA genes of the 2009 isolates differed from those of the 2008 isolates by substitution of two amino acids: I26T and Y40C (with the exception of A/Uganda/MUWRP-050/2008). The 2009 isolates clustered with the A/Victoria/208/09-like clade and the 2008 isolates clustered with the A/Brisbane/10/2007-like clade. No molecular marker of neuraminidase inhibitor resistance was observed in any of the isolates.

### Internal genes and gene products

Various amino acid mutations in the internal gene products differentiated the 2008 from the 2009 isolates, although some 2008 and 2009 isolates were differentiated only by one or more silent nucleotide mutations (data not shown) in the internal genes. The phyolgenetic relationships of the internal genes PB2, PB1, PA, NP, M, and NS in relation to vaccine reference strains are shown in Figure S1, Figure S2, Figure S3, Figure S4, Figure S5 and Figure S6, respectively. The aa positions of interest included drug resistance and increased virulence or enhanced transmission markers. All isolates had the S31N genetic marker for adamantane resistance in M2. Several molecular markers of enhanced transmission similar to other contemporary H3N2 influenza A viruses were detected in all isolates: A16G and C55F in M2, S409N in PA, and A199S, A661T, V667I, K702R and E627K (markers of increased virulence) in PB2. The PB2 D701, a marker of increased virulence was not observed in any of the isolates. The PB1-F2 protein of A/Uganda/MUWRP-007/2008 was truncated at position 28 (verified by re-sequencing the encoding gene twice) due to a cytosine-to thymine substitution at position 82. In addition, the PB1-F2 proteins of A/Uganda/MUWRP-062/2009 and A/Uganda/MUWRP-086/2009 were 3 amino acids shorter than those of the remaining 2008–2009 H3N2 strains.

## Discussion

We analyzed the coding sequences of all eight gene segments of influenza A/H3N2 isolates from the 2008 and 2009 influenza seasons in Uganda and compared them with those of the WHO-recommended vaccine strains for the 2009/10 and 2010/11 seasons [7] and with other epidemiologically relevant African strains from both GenBank and GISAID databases. To our knowledge, this is the first report of the full genomic sequencing of influenza viruses isolated in East Africa. We obtained the full genome sequence of 25 of the 59 Ugandan H3N2 isolates, with 59 full HA, M and NP; 56 full NA; 53 full NS; 44 full PA; 40 full PB1; and 36 full PB2 sequences. The similarity between Ugandan strains is such that the available information is sufficient to have an idea of the H3N2 virus circulation and evolution in the country and to compare these East African strains with viruses from around the world. We found that the 2008 and 2009 isolates were clearly differentiated by changes in all eight genome segments. Previous full-genome analyses of influenza viruses have identified striking differences between the phylogenetic clusters generated for different gene segments [15]. Most analyses of field isolates have used the HA1 domain of the HA gene because of its antigenic importance [2], [7]. However, viral fitness is affected not only by the antigenic properties of the HA gene but also by those of the NA gene and by the interaction of HA and NA with other viral proteins [23], each of which plays a significant role in promoting survival and replication.

The HA gene sequences indicated that all of the 2008 Ugandan isolates had evolved from A/Brisbane/10/07-like viruses; all of these viruses carried the signature genetic marker K173Q, and all of them remained within the A/Brisbane/10/07-like clade. The 2009 isolates appeared to have evolved away from the A/Brisbane/10/07-like viruses by acquiring the genetic markers N189K and K158N, which are characteristic of the A/Victoria/208/09 and A/Perth/16/09-like clades; these markers were reported in most other H3N2 influenza viruses isolated in Africa during that year and in many isolates globally. None of the Ugandan isolates clustered with the A/Perth/16/09-like subclade, which is defined by the additional substitutions E62K and N144K, but they all had the T212A substitution defining the A/Victoria/208/09 subclade [7]. A/Perth/16/09 is the recommended H3N2 reference virus for 2010–11 vaccines for both the northern and southern hemispheres and was shown to be antigenically similar to A/Victoria/208/09-like strains (WHO 2010). A number of non-Ugandan African isolates have clustered in the A/Perth/16/09 subclade. Although there was a clear differentiation between the 2008 and 2009 Ugandan isolates, some of the 2008 isolates clustered with viruses isolated in other parts of Africa in 2009.

The evolution of all of the Ugandan isolates from the A/Brisbane/10/07-like viruses was further confirmed by the presence of the signature genetic markers D147N and I125V in the NA gene [7]. The NA of influenza viruses plays a role in liberation of the viral progeny from infected cells and is a target of the neuraminidase inhibitors. Resistance to neuraminidase inhibitors is rare among influenza H3N2 isolates [24], and no markers of such resistance were found in our study.

All of the Ugandan isolates' internal genes contained markers that differentiated the 2008 and 2009 isolates, indicating a polygenic change between the two years. There was one unusual 2008 isolate (A/Uganda/MUWRP-050/2008) whose internal and NA genes showed many similarities to those of the 2009 isolates but whose HA gene did not. This isolate appeared to be an intermediate between the two seasonal strains. The adamantane resistance reported in most H3N2 viruses worldwide [25] was confirmed in the Ugandan isolates by an S31N substitution in the M2 protein. We found no significant changes in the M1 protein, which is reportedly involved in the generation of virus-like particles [26], [27] and is the most conserved influenza virus protein. At the nucleotide level, however, there was a silent C175A substitution that differentiated the 2008 and 2009 isolates.

The RNA polymerase genes PB1, PB2, and PA are involved in both transcription and replication of the genome. The PB1 gene encodes an RNA polymerase and a PB1-F2 protein from an alternative open reading frame [27]. In all of the 2009 isolates, the PB1-F2 protein had the three amino acid substitutions E4G, I16T, and N34S, all of which were also reported in human seasonal H3N2 isolates in Thailand [28]. These changes were also found in one 2008 isolate, A/Uganda/MUWRP-050/2008. The effect of these mutations on viral fitness is unknown. The N66S substitution, which is the only PB1-F2–associated genetic marker of increased pathogenicity, was not identified. One isolate (A/Uganda/MUWRP-007/2008) had a truncated, nonfunctional PB1-F2 protein due to a single nucleotide substitution at position 28. It is suggested that PB1-F2 is not expressed in all influenza strains, but it is known to play a role in pathogenicity by inducing apoptosis and exacerbating pro-inflammatory effects [29]. A truncated PB1-F2 protein has also been reported in other strains, including the 2009 pandemic H1N1 strain. Further phenotypic studies are required to determine whether A/Uganda/MUWRP-007/2008 is less virulent than co-circulating H3N2 strains because of this PB1-F2 truncation. All of the Ugandan isolates had the L13P substitution in the PB1 protein, which enhances polymerase activity and increases virulence by improving PA polymerase affinity [30], and the I317M substitution in the PB1 protein, which is reportedly related to increased virulence in mice [31].

The NP gene segments of the studied isolates did not contain the markers (L136M, N319K) of enhanced transmission. Non-structural proteins are not components of the viral particles but are expressed at high levels in infected cells; the T92E substitution in the NS1 protein has been implicated in pathogenicity and severity of disease [27], [24].

Overall, the Ugandan influenza A/H3N2 isolates from the 2008 and 2009 influenza seasons phylogenetically clustered separately. They carried signature markers of the previous vaccine strain A/Brisbane/10/07 but had continued to evolve and changes could be observed in all gene segments. The 2009 isolates clustered with the A/Victoria/208/09 clade and shared some properties with the A/Perth/16/09. Close follow-up of shifts during future seasons. Genetic and antigenic drift is responsible for the diversity seen in contemporary seasonal H3N2. But full genome sequencing can be used for monitoring of reassortment events within H3N2 and between other Flu A which is vital to ensure that selected vaccine strains are protective against the strains circulating in this region [32]. Despite the increasing availability of whole influenza genome sequences in the developed world, this is the second report to our knowledge of an analysis of the complete genome sequence of African influenza isolates and the first such report from East Africa. This data will be very valuable in tracking the phylogenetic evolution of influenza viruses within sub-Saharan Africa.

## Supporting Information

Figure S1 **Phylogenetic analysis of the influenza A/H3N2 PB2 genes.** Most Ugandan 2008–2009 PB2 gene sequences were identical to A/Uganda/MUWRP-01/2008 at the amino acid level except for the cluster of A/Uganda/MUWRP-50/2008 identical to A/Uganda/MUWRP-58/2009, A/Uganda/MUWRP-62/2009, A/Uganda/MUWRP-75/2009, A/Uganda/MUWRP-79/2009, and A/Uganda/MUWRP-86/2009; and the following unique sequences present on the tree: A/Uganda/MUWRP-6/2008, A/Uganda/MUWRP-8/2008, A/Uganda/MUWRP-13/2008, A/Uganda/MUWRP-14/2008, A/Uganda/MUWRP-15/2008, A/Uganda/MUWRP-17/2008, A/Uganda/MUWRP-19/2008, A/Uganda/MUWRP-20/2008, A/Uganda/MUWRP-21/2008, A/Uganda/MUWRP-24/2008, A/Uganda/MUWRP-27/2008, A/Uganda/MUWRP-28/2008, A/Uganda/MUWRP-35/2008, A/Uganda/MUWRP-36/2008, A/Uganda/MUWRP-37/2008, A/Uganda/MUWRP-38/2008, A/Uganda/MUWRP-39/2008, A/Uganda/MUWRP-40/2008, A/Uganda/MUWRP-41/2008, A/Uganda/MUWRP-42/2008, A/Uganda/MUWRP-44/2008, A/Uganda/MUWRP-45/2008, A/Uganda/MUWRP-48/2008, A/Uganda/MUWRP-52/2008, A/Uganda/MUWRP-61/2009, A/Uganda/MUWRP-65/2009, A/Uganda/MUWRP-70/2009, and A/Uganda/MUWRP-74/2009. Our Ugandan PB2 gene sequences (in bold) were compared to the 2 available vaccine strains A/Wisconsin/67/2005 and A/Brisbane/10/2007 (in italic and underlined) and all available African strains (only 2 sequences from Dakar, Senegal). Bootstrap values >49 are indicated at the tree's nodes.
(TIF)

Figure S2 **Phylogenetic analysis of the influenza A/H3N2 PB1 genes.** Ugandan 2008–2009 PB1 gene sequences identical to A/Uganda/MUWRP-01/2008 at the amino acid level were excluded from the tree: A/Uganda/MUWRP-02/2008, A/Uganda/MUWRP-05/2008, A/Uganda/MUWRP-06/2008, A/Uganda/MUWRP-08/2008, A/Uganda/MUWRP-09/2008, A/Uganda/MUWRP-13/2008, A/Uganda/MUWRP-14/2008, A/Uganda/MUWRP-16/2008, A/Uganda/MUWRP-17/2008, A/Uganda/MUWRP-24/2008, A/Uganda/MUWRP-26/2008, A/Uganda/MUWRP-28/2008, A/Uganda/MUWRP-29/2008, A/Uganda/MUWRP-32/2008, A/Uganda/MUWRP-46/2008, A/Uganda/MUWRP-47/2008, A/Uganda/MUWRP-48/2008, A/Uganda/MUWRP-49/2008, and A/Uganda/MUWRP-51/2008; as were A/Uganda/MUWRP-58/2009, A/Uganda/MUWRP-61/2009, A/Uganda/MUWRP-65/2009 and A/Uganda/MUWRP-75/2009 identical to A/Uganda/MUWRP-50/2008; and A/Uganda/MUWRP-31/2008 and A/Uganda/MUWRP-33/2008 identical to A/Uganda/MUWRP-15/2008. Our Ugandan PB1 gene sequences (in bold) were compared to the 2 available vaccine strains A/Wisconsin/67/2005 and A/Brisbane/10/2007 (in italic and underlined) and all available African strains (only 2 sequences from Dakar, Senegal). Bootstrap values >49 are indicated at the tree's nodes.
(TIF)

Figure S3 **Phylogenetic analysis of the influenza A/H3N2 PA genes.** Ugandan 2008–2009 PA gene sequences identical to A/Uganda/MUWRP-01/2008 at the amino acid level were excluded from the tree: A/Uganda/MUWRP-03/2008, A/Uganda/MUWRP-05/2008, A/Uganda/MUWRP-07/2008, A/Uganda/MUWRP-08/2008, A/Uganda/MUWRP-10/2008, A/Uganda/MUWRP-11/2008, A/Uganda/MUWRP-16/2008, A/Uganda/MUWRP-17/2008, A/Uganda/MUWRP-23/2008, A/Uganda/MUWRP-27/2008, A/Uganda/MUWRP-31/2008, A/Uganda/MUWRP-33/2008, A/Uganda/MUWRP-38/2008, A/Uganda/MUWRP-40/2008, A/Uganda/MUWRP-41/2008, A/Uganda/MUWRP-42/2008, A/Uganda/MUWRP-43/2008, and A/Uganda/MUWRP-45/2008; as was A/Uganda/MUWRP-46/2008 identical to A/Uganda/MUWRP-47/2008; A/Uganda/MUWRP-86/2009 identical to A/Uganda/MUWRP-61/2009; and A/Uganda/MUWRP-58/2009, A/Uganda/MUWRP-65/2009, A/Uganda/MUWRP-74/2009, A/Uganda/MUWRP-75/2009, and A/Uganda/MUWRP-79/2009 identical to A/Uganda/MUWRP-50/2009. Our Ugandan PA gene sequences (in bold) were compared to the 2 available vaccine strains A/Wisconsin/67/2005 and A/Brisbane/10/2007 (in italic and underlined) and all available African strains (only 2 sequences from Dakar, Senegal). Bootstrap values >49 are indicated at the tree's nodes.
(TIFF)

Figure S4 **Phylogenetic analysis of the influenza A/H3N2 NP genes.** Ugandan 2008–2009 NP gene sequences were all identical to A/Uganda/MUWRP-01/2008 at the amino acid level except for A/Uganda/MUWRP-03/2008, A/Uganda/MUWRP-05/2008, A/Uganda/MUWRP-16/2008, and A/Uganda/MUWRP-23/2008 = A/Uganda/MUWRP-45/2008, which is why only 4 strains appear on the phylogenetic tree. Our Ugandan NP gene sequences (in bold) were compared to the 2 available vaccine strains A/Wisconsin/67/2005 and A/Brisbane/10/2007 (in italic and underlined) and all available African strains (only 2 sequences from Dakar, Senegal). Bootstrap values >49 are indicated at the tree's nodes.
(TIF)

Figure S5 **Phylogenetic analysis of the influenza A/H3N2 M genes.** Most Ugandan 2008–2009 M gene sequences were identical to A/Uganda/MUWRP-01/2008 at the amino acid level except for the cluster of A/Uganda/MUWRP-61/2009 identical to A/Uganda/MUWRP-65/2009, A/Uganda/MUWRP-74/2009, A/Uganda/MUWRP-75/2009, A/Uganda/MUWRP-79/2009, and A/Uganda/MUWRP-86/2009; A/Uganda/MUWRP-50/2008 identical to A/Uganda/MUWRP-58/2009; and the following unique sequences present on the tree: A/Uganda/MUWRP-16/2008, A/Uganda/MUWRP-18/2008, A/Uganda/MUWRP-20/2008, A/Uganda/MUWRP-26/2008, A/Uganda/MUWRP-36/2008, and A/Uganda/MUWRP-70/2009. Our Ugandan M gene sequences (in bold) were compared to the 2 available vaccine strains A/Wisconsin/67/2005 and A/Brisbane/10/2007 (in italic and underlined) and all available African strains (identical sequences were removed from the analysis keeping a single representing virus per country). Bootstrap values >49 are indicated at the tree's nodes.
(TIF)

Figure S6 **Phylogenetic analysis of the influenza A/H3N2 NS genes.** Ugandan 2008–2009 NS gene sequences identical to A/Uganda/MUWRP-01/2008 at the amino acid level were excluded from the tree: A/Uganda/MUWRP-06/2008, A/Uganda/MUWRP-22/2008, A/Uganda/MUWRP-25/2008, A/Uganda/MUWRP-29/2008, A/Uganda/MUWRP-34/2008, A/Uganda/MUWRP-36/2008, A/Uganda/MUWRP-40/2008, A/Uganda/MUWRP-42/2008, A/Uganda/MUWRP-43/2008, A/Uganda/MUWRP-44/2008, A/Uganda/MUWRP-48/2008, and A/Uganda/MUWRP-52/2008; as were A/Uganda/MUWRP-8/2008, A/Uganda/MUWRP-9/2008, A/Uganda/MUWRP-10/2008, A/Uganda/MUWRP-13/2008, A/Uganda/MUWRP-14/2008, A/Uganda/MUWRP-17/2008, A/Uganda/MUWRP-18/2008, A/Uganda/MUWRP-19/2008, A/Uganda/MUWRP-21/2008, A/Uganda/MUWRP-27/2008, A/Uganda/MUWRP-28/2008, and A/Uganda/MUWRP-51/2008 identical to A/Uganda/MUWRP-3/2008; A/Uganda/MUWRP-20/2008, A/Uganda/MUWRP-32/2008, A/Uganda/MUWRP-38/2009, and A/Uganda/MUWRP-41/2008 identical to A/Uganda/MUWRP-11/2008; A/Uganda/MUWRP-31/2008, A/Uganda/MUWRP-33/2008, and A/Uganda/MUWRP-39/2008 to A/Uganda/MUWRP-15/2008; A/Uganda/MUWRP-23/2008 to A/Uganda/MUWRP-45/2008; and A/Uganda/MUWRP-61/2009, A/Uganda/MUWRP-65/2009, A/Uganda/MUWRP-70/2009, A/Uganda/MUWRP-74/2009, A/Uganda/MUWRP-75/2009, and A/Uganda/MUWRP-79/2009 to A/Uganda/MUWRP-58/2009. Our Ugandan NS gene sequences (in bold) were compared to the 2 available vaccine strains A/Wisconsin/67/2005 and A/Brisbane/10/2007 (in italic and underlined) and all available African strains (only 2 sequences from Dakar, Senegal). Bootstrap values >49 are indicated at the tree's nodes.
(TIF)
