# Supplementary figures and images for: Molecular Epidemiology of Influenza A/H3N2 Viruses Circulating in Uganda
Source: PLoS One. 2011 Nov 21;6(11):e27803. doi: 10.1371/journal.pone.0027803 (PMC3221667; doi:10.1371/journal.pone.0027803)

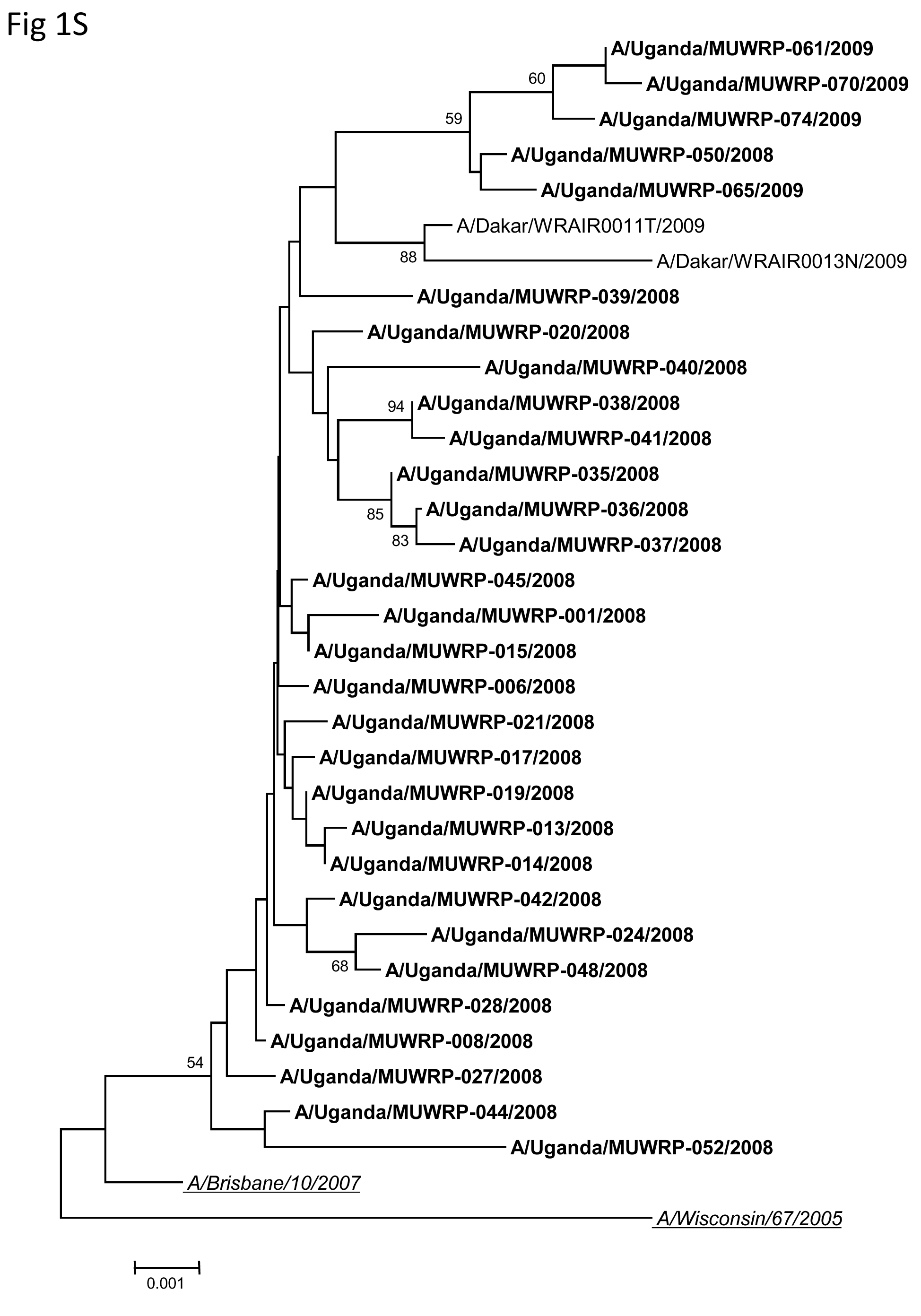

Supplement: Figure S1 — Phylogenetic analysis of the influenza A/H3N2 PB2 genes. Most Ugandan 2008–2009 PB2 gene sequences were identical to A/Uganda/MUWRP-01/2008 at the amino acid level except for the cluster of A/Uganda/MUWRP-50/2008 identical to A/Uganda/MUWRP-58/2009, A/Uganda/MUWRP-62/2009, A/Uganda/MUWRP-75/2009, A/Uganda/MUWRP-79/2009, and A/Uganda/MUWRP-86/2009; and the following unique sequences present on the tree: A/Uganda/MUWRP-6/2008, A/Uganda/MUWRP-8/2008, A/Uganda/MUWRP-13/2008, A/Uganda/MUWRP-14/2008, A/Uganda/MUWRP-15/2008, A/Uganda/MUWRP-17/2008, A/Uganda/MUWRP-19/2008, A/Uganda/MUWRP-20/2008, A/Uganda/MUWRP-21/2008, A/Uganda/MUWRP-24/2008, A/Uganda/MUWRP-27/2008, A/Uganda/MUWRP-28/2008, A/Uganda/MUWRP-35/2008, A/Uganda/MUWRP-36/2008, A/Uganda/MUWRP-37/2008, A/Uganda/MUWRP-38/2008, A/Uganda/MUWRP-39/2008, A/Uganda/MUWRP-40/2008, A/Uganda/MUWRP-41/2008, A/Uganda/MUWRP-42/2008, A/Uganda/MUWRP-44/2008, A/Uganda/MUWRP-45/2008, A/Uganda/MUWRP-48/2008, A/Uganda/MUWRP-52/2008, A/Uganda/MUWRP-61/2009, A/Uganda/MUWRP-65/2009, A/Uganda/MUWRP-70/2009, and A/Uganda/MUWRP-74/2009. Our Ugandan PB2 gene sequences (in bold) were compared to the 2 available vaccine strains A/Wisconsin/67/2005 and A/Brisbane/10/2007 (in italic and underlined) and all available African strains (only 2 sequences from Dakar, Senegal). Bootstrap values >49 are indicated at the tree's nodes. (TIF) [file pone.0027803.s001.tif]

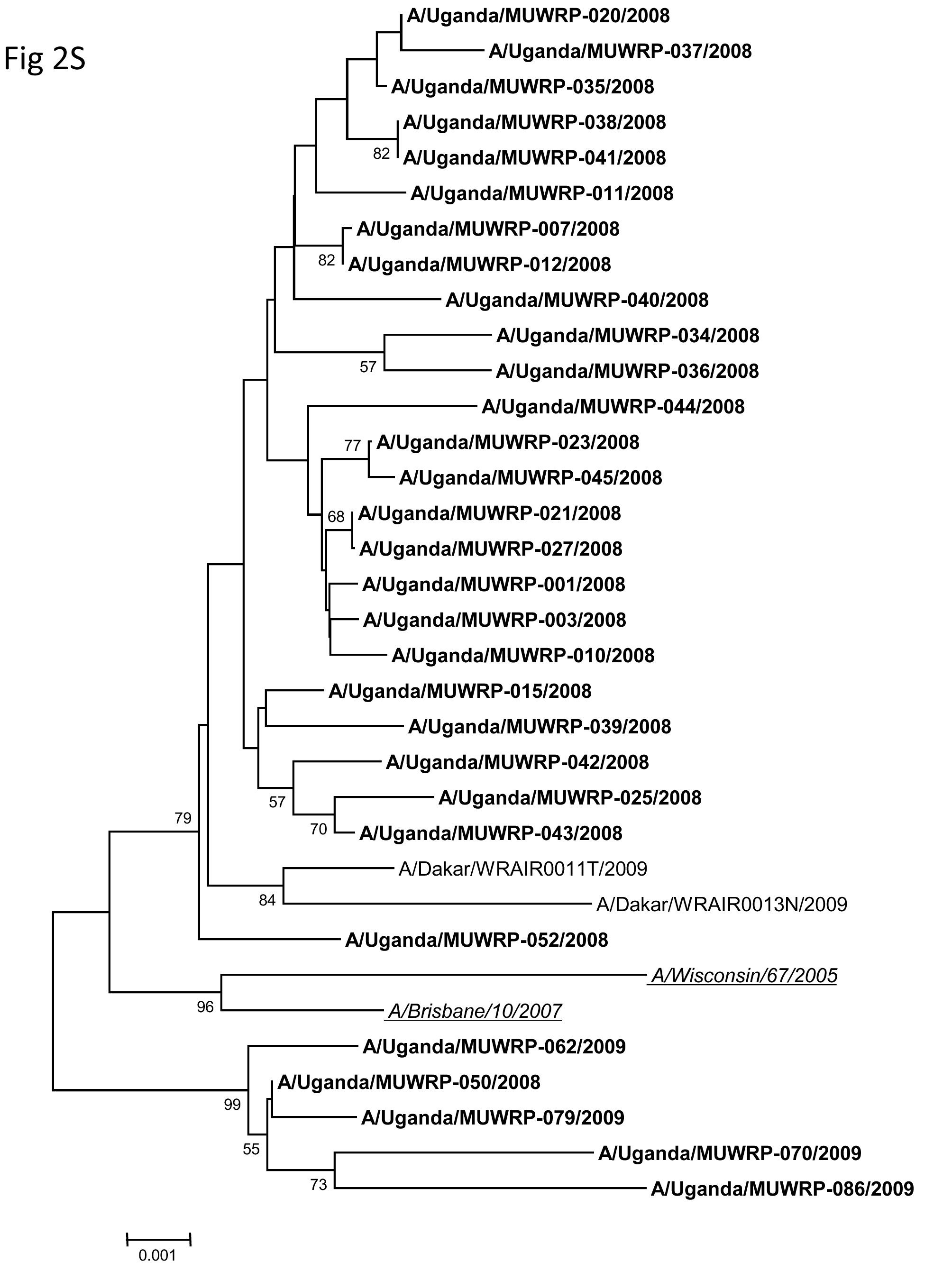

Supplement: Figure S2 — Phylogenetic analysis of the influenza A/H3N2 PB1 genes. Ugandan 2008–2009 PB1 gene sequences identical to A/Uganda/MUWRP-01/2008 at the amino acid level were excluded from the tree: A/Uganda/MUWRP-02/2008, A/Uganda/MUWRP-05/2008, A/Uganda/MUWRP-06/2008, A/Uganda/MUWRP-08/2008, A/Uganda/MUWRP-09/2008, A/Uganda/MUWRP-13/2008, A/Uganda/MUWRP-14/2008, A/Uganda/MUWRP-16/2008, A/Uganda/MUWRP-17/2008, A/Uganda/MUWRP-24/2008, A/Uganda/MUWRP-26/2008, A/Uganda/MUWRP-28/2008, A/Uganda/MUWRP-29/2008, A/Uganda/MUWRP-32/2008, A/Uganda/MUWRP-46/2008, A/Uganda/MUWRP-47/2008, A/Uganda/MUWRP-48/2008, A/Uganda/MUWRP-49/2008, and A/Uganda/MUWRP-51/2008; as were A/Uganda/MUWRP-58/2009, A/Uganda/MUWRP-61/2009, A/Uganda/MUWRP-65/2009 and A/Uganda/MUWRP-75/2009 identical to A/Uganda/MUWRP-50/2008; and A/Uganda/MUWRP-31/2008 and A/Uganda/MUWRP-33/2008 identical to A/Uganda/MUWRP-15/2008. Our Ugandan PB1 gene sequences (in bold) were compared to the 2 available vaccine strains A/Wisconsin/67/2005 and A/Brisbane/10/2007 (in italic and underlined) and all available African strains (only 2 sequences from Dakar, Senegal). Bootstrap values >49 are indicated at the tree's nodes. (TIF) [file pone.0027803.s002.tif]

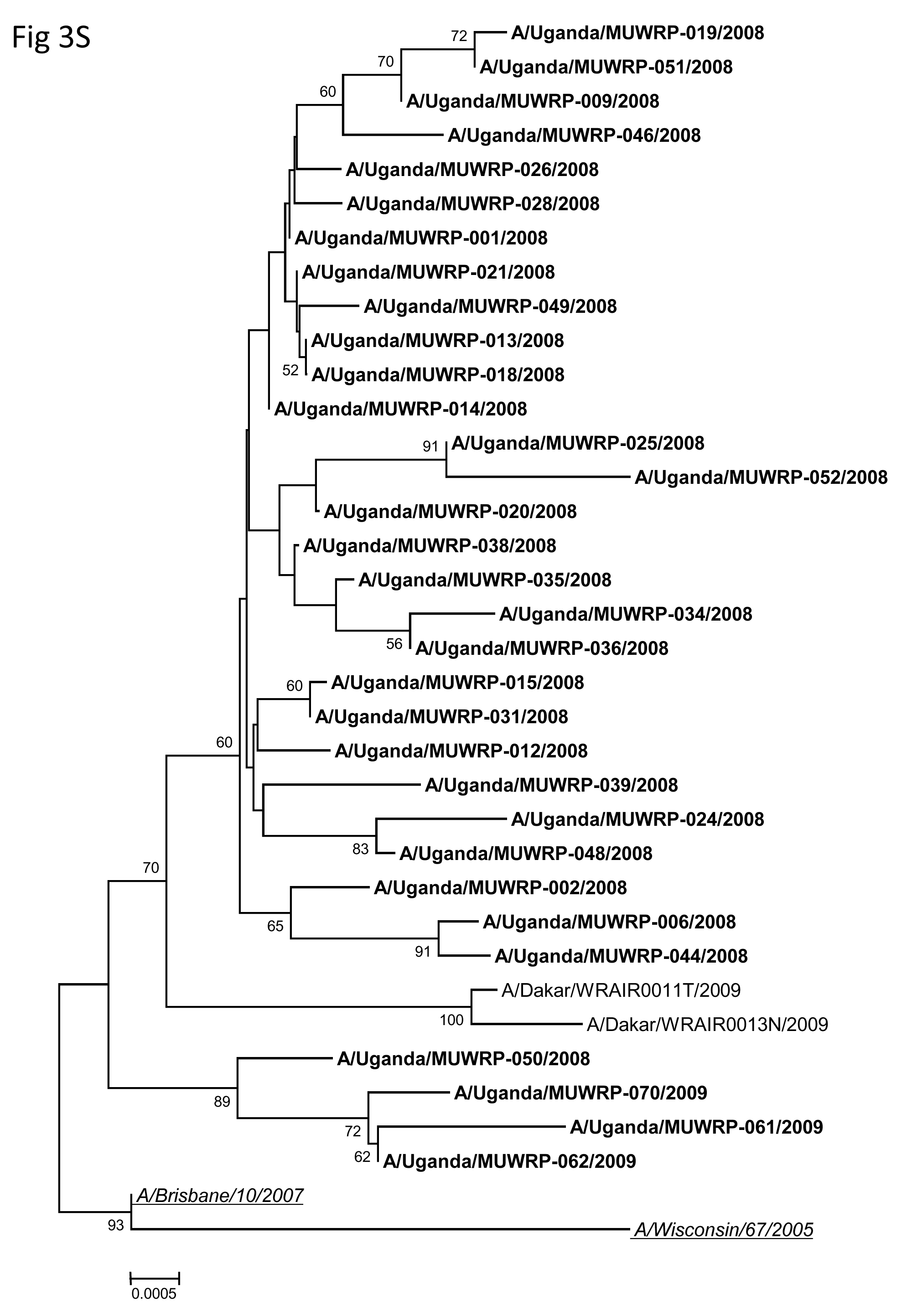

Supplement: Figure S3 — Phylogenetic analysis of the influenza A/H3N2 PA genes. Ugandan 2008–2009 PA gene sequences identical to A/Uganda/MUWRP-01/2008 at the amino acid level were excluded from the tree: A/Uganda/MUWRP-03/2008, A/Uganda/MUWRP-05/2008, A/Uganda/MUWRP-07/2008, A/Uganda/MUWRP-08/2008, A/Uganda/MUWRP-10/2008, A/Uganda/MUWRP-11/2008, A/Uganda/MUWRP-16/2008, A/Uganda/MUWRP-17/2008, A/Uganda/MUWRP-23/2008, A/Uganda/MUWRP-27/2008, A/Uganda/MUWRP-31/2008, A/Uganda/MUWRP-33/2008, A/Uganda/MUWRP-38/2008, A/Uganda/MUWRP-40/2008, A/Uganda/MUWRP-41/2008, A/Uganda/MUWRP-42/2008, A/Uganda/MUWRP-43/2008, and A/Uganda/MUWRP-45/2008; as was A/Uganda/MUWRP-46/2008 identical to A/Uganda/MUWRP-47/2008; A/Uganda/MUWRP-86/2009 identical to A/Uganda/MUWRP-61/2009; and A/Uganda/MUWRP-58/2009, A/Uganda/MUWRP-65/2009, A/Uganda/MUWRP-74/2009, A/Uganda/MUWRP-75/2009, and A/Uganda/MUWRP-79/2009 identical to A/Uganda/MUWRP-50/2009. Our Ugandan PA gene sequences (in bold) were compared to the 2 available vaccine strains A/Wisconsin/67/2005 and A/Brisbane/10/2007 (in italic and underlined) and all available African strains (only 2 sequences from Dakar, Senegal). Bootstrap values >49 are indicated at the tree's nodes. (TIFF) [file pone.0027803.s003.tiff]

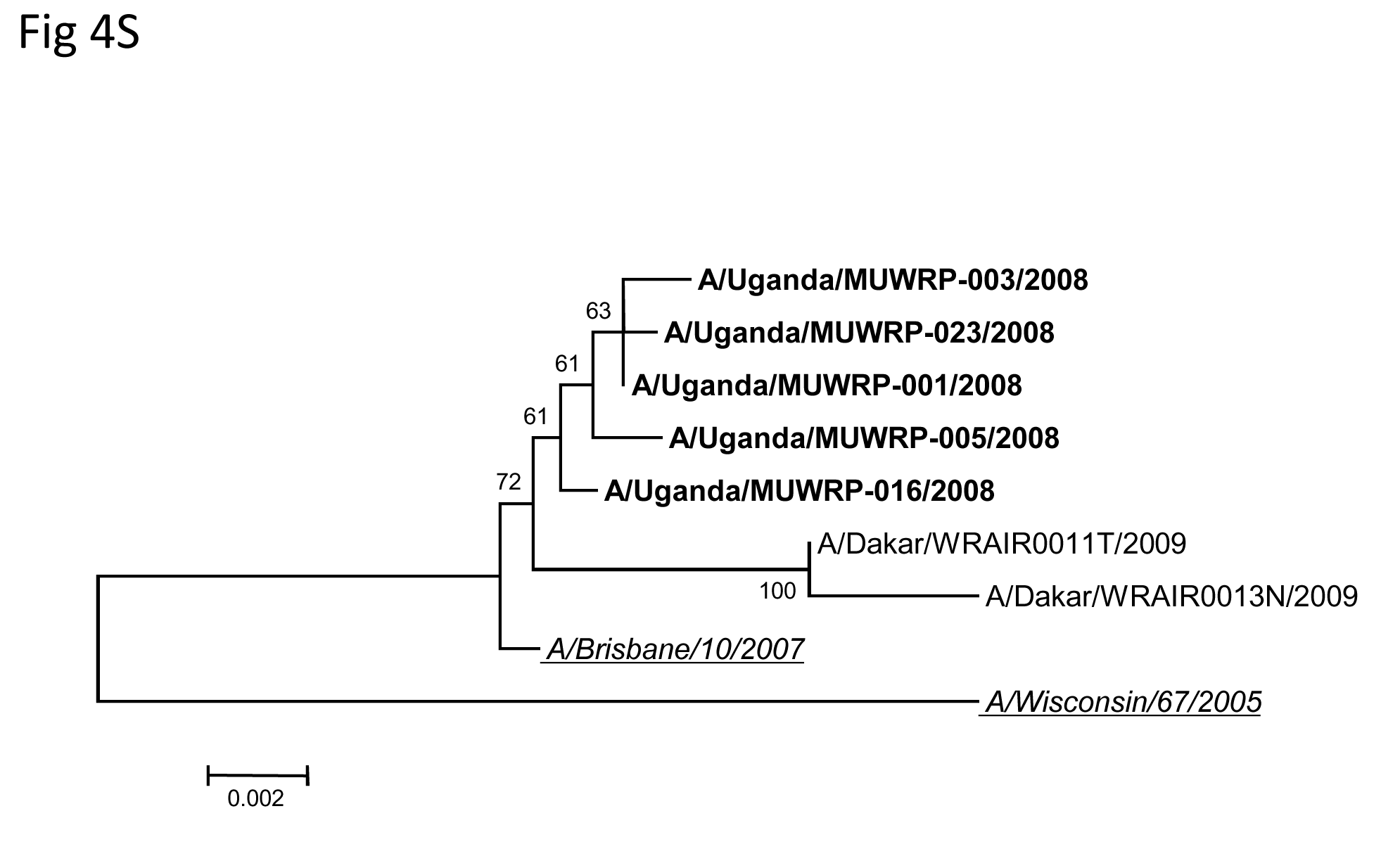

Supplement: Figure S4 — Phylogenetic analysis of the influenza A/H3N2 NP genes. Ugandan 2008–2009 NP gene sequences were all identical to A/Uganda/MUWRP-01/2008 at the amino acid level except for A/Uganda/MUWRP-03/2008, A/Uganda/MUWRP-05/2008, A/Uganda/MUWRP-16/2008, and A/Uganda/MUWRP-23/2008 = A/Uganda/MUWRP-45/2008, which is why only 4 strains appear on the phylogenetic tree. Our Ugandan NP gene sequences (in bold) were compared to the 2 available vaccine strains A/Wisconsin/67/2005 and A/Brisbane/10/2007 (in italic and underlined) and all available African strains (only 2 sequences from Dakar, Senegal). Bootstrap values >49 are indicated at the tree's nodes. (TIF) [file pone.0027803.s004.tif]

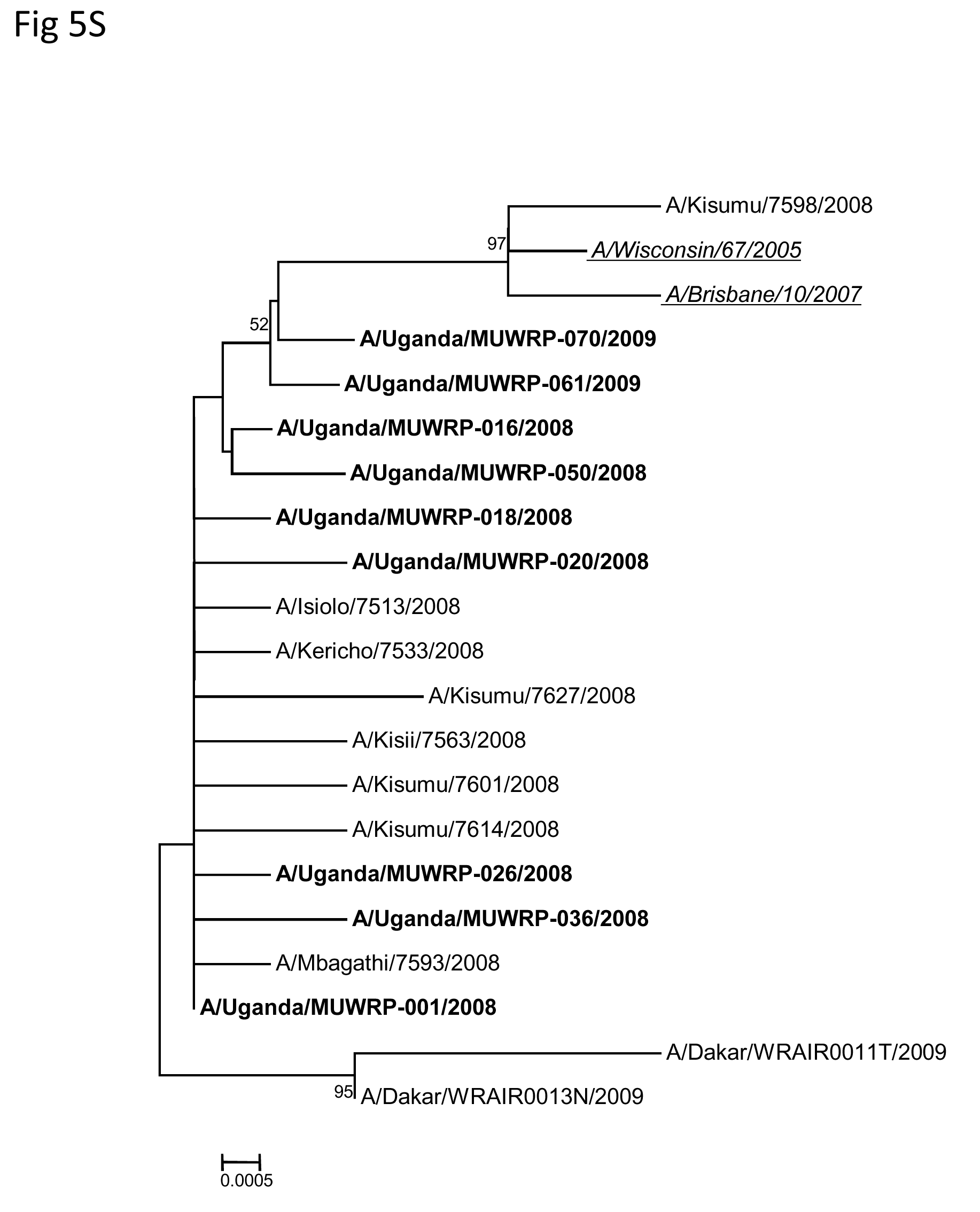

Supplement: Figure S5 — Phylogenetic analysis of the influenza A/H3N2 M genes. Most Ugandan 2008–2009 M gene sequences were identical to A/Uganda/MUWRP-01/2008 at the amino acid level except for the cluster of A/Uganda/MUWRP-61/2009 identical to A/Uganda/MUWRP-65/2009, A/Uganda/MUWRP-74/2009, A/Uganda/MUWRP-75/2009, A/Uganda/MUWRP-79/2009, and A/Uganda/MUWRP-86/2009; A/Uganda/MUWRP-50/2008 identical to A/Uganda/MUWRP-58/2009; and the following unique sequences present on the tree: A/Uganda/MUWRP-16/2008, A/Uganda/MUWRP-18/2008, A/Uganda/MUWRP-20/2008, A/Uganda/MUWRP-26/2008, A/Uganda/MUWRP-36/2008, and A/Uganda/MUWRP-70/2009. Our Ugandan M gene sequences (in bold) were compared to the 2 available vaccine strains A/Wisconsin/67/2005 and A/Brisbane/10/2007 (in italic and underlined) and all available African strains (identical sequences were removed from the analysis keeping a single representing virus per country). Bootstrap values >49 are indicated at the tree's nodes. (TIF) [file pone.0027803.s005.tif]

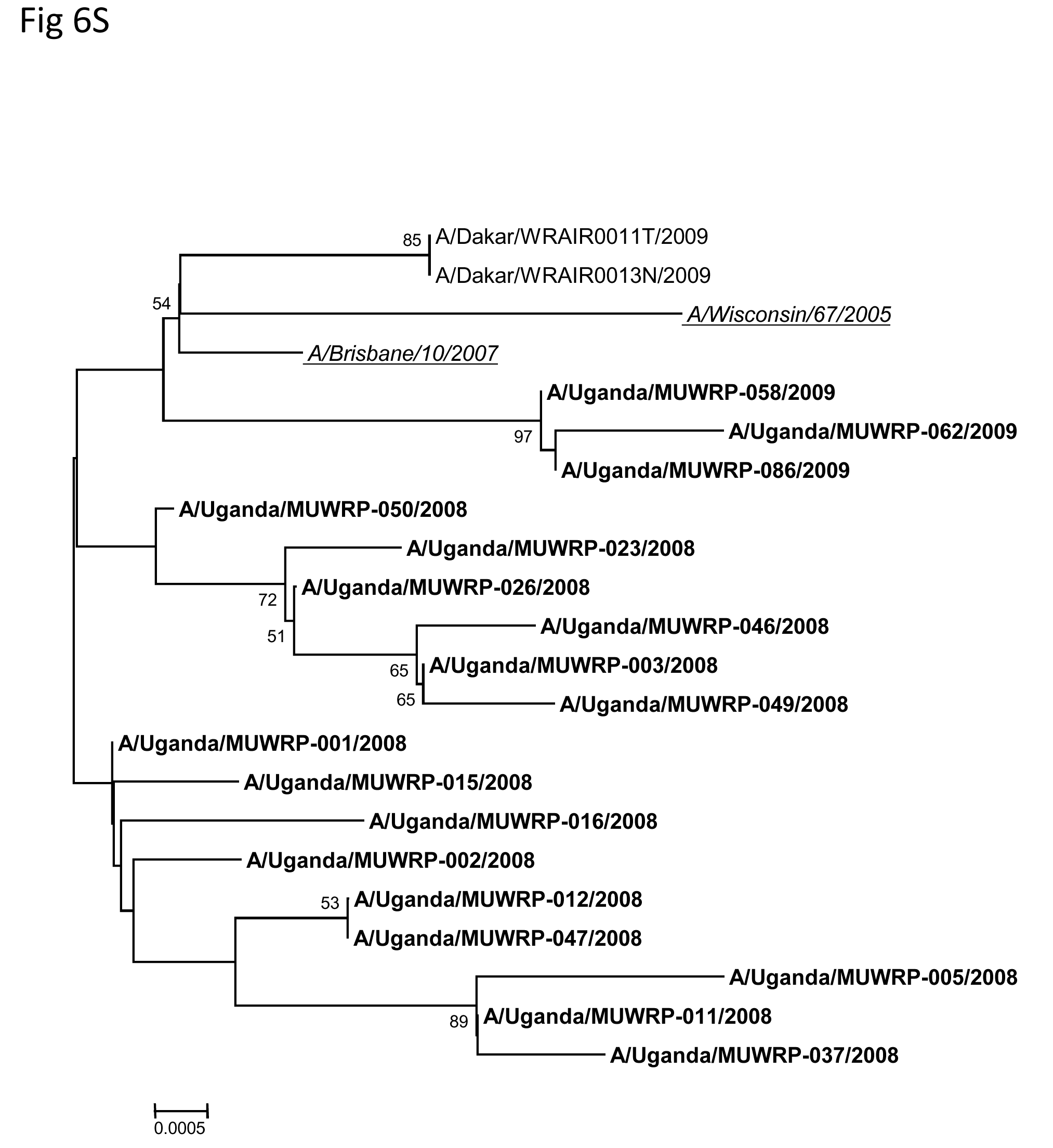

Supplement: Figure S6 — Phylogenetic analysis of the influenza A/H3N2 NS genes. Ugandan 2008–2009 NS gene sequences identical to A/Uganda/MUWRP-01/2008 at the amino acid level were excluded from the tree: A/Uganda/MUWRP-06/2008, A/Uganda/MUWRP-22/2008, A/Uganda/MUWRP-25/2008, A/Uganda/MUWRP-29/2008, A/Uganda/MUWRP-34/2008, A/Uganda/MUWRP-36/2008, A/Uganda/MUWRP-40/2008, A/Uganda/MUWRP-42/2008, A/Uganda/MUWRP-43/2008, A/Uganda/MUWRP-44/2008, A/Uganda/MUWRP-48/2008, and A/Uganda/MUWRP-52/2008; as were A/Uganda/MUWRP-8/2008, A/Uganda/MUWRP-9/2008, A/Uganda/MUWRP-10/2008, A/Uganda/MUWRP-13/2008, A/Uganda/MUWRP-14/2008, A/Uganda/MUWRP-17/2008, A/Uganda/MUWRP-18/2008, A/Uganda/MUWRP-19/2008, A/Uganda/MUWRP-21/2008, A/Uganda/MUWRP-27/2008, A/Uganda/MUWRP-28/2008, and A/Uganda/MUWRP-51/2008 identical to A/Uganda/MUWRP-3/2008; A/Uganda/MUWRP-20/2008, A/Uganda/MUWRP-32/2008, A/Uganda/MUWRP-38/2009, and A/Uganda/MUWRP-41/2008 identical to A/Uganda/MUWRP-11/2008; A/Uganda/MUWRP-31/2008, A/Uganda/MUWRP-33/2008, and A/Uganda/MUWRP-39/2008 to A/Uganda/MUWRP-15/2008; A/Uganda/MUWRP-23/2008 to A/Uganda/MUWRP-45/2008; and A/Uganda/MUWRP-61/2009, A/Uganda/MUWRP-65/2009, A/Uganda/MUWRP-70/2009, A/Uganda/MUWRP-74/2009, A/Uganda/MUWRP-75/2009, and A/Uganda/MUWRP-79/2009 to A/Uganda/MUWRP-58/2009. Our Ugandan NS gene sequences (in bold) were compared to the 2 available vaccine strains A/Wisconsin/67/2005 and A/Brisbane/10/2007 (in italic and underlined) and all available African strains (only 2 sequences from Dakar, Senegal). Bootstrap values >49 are indicated at the tree's nodes. (TIF) [file pone.0027803.s006.tif]
